# Understanding who is and isn’t involved and engaged in health research: capturing and analysing demographic data to diversify patient and public involvement and engagement

**DOI:** 10.1186/s40900-023-00434-5

**Published:** 2023-05-08

**Authors:** Annie Keane, Safina Islam, Suzanne Parsons, Arpana Verma, Tracey Farragher, Davine Forde, Leah Holmes, Katharine Cresswell, Susannah Williams, Paolo Arru, Emily Howlett, Hannah Turner-Uaandja, Issy MacGregor, Tracy Grey, Zahra Arain, Maura Scahill, Bella Starling

**Affiliations:** 1 Vocal, NIHR Manchester Biomedical Research Centre, Manchester Academic Health Sciences Centre, Manchester University NHS Foundation Trust, The Nowgen Centre, Grafton Street, Manchester, M13 9WU UK; 2 Ahmed Iqbal Ullah Race Relations Resource Centre and Education Trust (Previously at Vocal), University of Manchester, Manchester, UK; 3 Vocal, NIHR Manchester Biomedical Research Centre, Manchester Academic Health Sciences Centre, NIHR Clinical Research Facility, Manchester University NHS Foundation Trust, Manchester, UK; 4 Division of Population Health, Health Services Research and Primary Care and Manchester Urban Collaboration, University of Manchester, Manchester, UK; 5 Division of Population Health, Health Services Research and Primary Care, University of Manchester, Manchester, UK; 6 Manchester BME Network CIC (Public Contributor and Community Partner), Manchester, UK; 7 Science Policy and Research Programme (Previously at Vocal), National Institute for Health and Care Excellence, City Tower, Piccadilly Plaza, Manchester, M1 4BT UK

**Keywords:** Patient and public involvement and engagement (PPIE), Health inequalities, Protected characteristics, Demographics, Equality, diversity and inclusion (EDI), Inclusive research, inclusion

## Abstract

**Background:**

Patient and public involvement and engagement (PPIE) can improve the relevance, quality, ethics and impact of research thus contributing to high quality research. Currently in the UK, people who get involved in research tend to be aged 61 years or above, White and female. Calls for greater diversity and inclusion in PPIE have become more urgent especially since the COVID-19 pandemic, so that research can better address health inequalities and be relevant for all sectors of society. Yet, there are currently no routine systems or requirements to collect or analyse the demographics of people who get involved in health research in the UK. The aim of this study was to develop to capture and analyse the characteristics of who does and doesn’t take part in patient and public involvement and engagement (PPIE) activities.

**Methods:**

As part of its strategic focus on diversity and inclusion, Vocal developed a questionnaire to assess the demographics of people taking part in its PPIE activities. Vocal is a non-profit organisation which supports PPIE in health research across the region of Greater Manchester in England. The questionnaire was implemented across Vocal activities between December 2018 and March 2022. In that time. Vocal was working with approximately 935 public contributors. 329 responses were received: a return rate of 29.3%. Analysis of findings and comparison against local population demographic data, and available national data related to public contributors to health research, was performed.

**Results:**

Results show that it is feasible to assess the demographics of people who take part in PPIE activities, through a questionnaire system. Further, our emerging data indicate that Vocal are involving people from a wider range of ages and with a greater diversity of ethnic backgrounds in health research, as compared to available national data. Specifically, Vocal involves more people of Asian, African and Caribbean heritage, and includes a wider range of ages in its PPIE activities. More women than men are involved in Vocal’s work.

**Conclusion:**

Our ‘learn by doing’ approach to assessing who does and doesn’t take part in Vocal’s PPIE activities has informed our practice and continues influence our strategic priorities for PPIE. Our system and learning reported here may be applicable and transferable to other similar settings in which PPIE is carried out. We attribute the greater diversity of our public contributors to our strategic priority and activities to promote more inclusive research since 2018.

## Abstract (summary)

Patient and public involvement and engagement (PPIE) can improve the relevance, quality, ethics and impact of research thus contributing to high quality research. Currently in the UK, people who get involved in research tend to be aged 61 years or above, White and female. Calls for greater diversity and inclusion in PPIE have become more urgent especially since the COVID-19 pandemic, so that research can better address health inequalities and be relevant for all sectors of society. Yet, there are currently no routine systems or requirements to collect or analyse the demographics of people who get involved in health research in the UK. Vocal is a non-profit organisation which supports PPIE in health research across the region of Greater Manchester in England. Since 2018, one of Vocal’s strategic priorities has been to promote inclusive research by diversifying those who are engaged and involved in research, through the development of more inclusive ways of working together, including methods to understand who is (and isn’t) currently involved in Vocal’s PPIE activities. We find that it’s feasible to capture and analyse demographic data related to PPIE. Further, our emerging data indicate that we are involving people from a wider range of ages and with a greater diversity of ethnic backgrounds in PPIE for health research, as compared to available national data. However, similarly to national trends, more women than men are involved in PPIE work.

## Introduction

In recent years, issues of health equity and clinical research have been brought into sharp focus and particularly highlighted during the COVID-19 pandemic. High quality research, including early phase, experimental medicine and clinical trials, are the basis for evidence-based healthcare and can change lives. High quality research requires inclusive and diverse patient and public involvement and engagement (PPIE) in order to be generalisable, benefit all in society, be relevant to patient needs, and address health inequalities [1, 2]. For clarity, we define PPIE as an active collaborative partnership between researchers and members of the public, patients, carers and/or communities, working alongside research teams and as part of research organisations.

PPIE is becoming an established feature of high quality health research in the United Kingdom (UK) and elsewhere and is expected by many funders. PPIE can ensure that research is prioritised and designed to meet the needs of those who might benefit the most from its results and is key to the relevance, applicability and uptake of high quality research [2, 3]. Researchers that engage with PPIE gain new skills and changes in attitudes and priorities because of the knowledge and experience of working with people with different perspectives [4]. There is evidence to suggest that PPIE also increases the recruitment and retention of participants to clinical trials [5].

PPIE is not automatically inclusive but inclusive PPIE is an important mechanism for increasing diversity and addressing health inequalities in research. Evidence shows that people from deprived areas are more likely to experience poorer health outcomes [6] and they are also less likely to be recruited to health related research [7]. By seeking the input of more diverse lived experience, PPIE has the potential to contribute to more inclusive research, relevant to health need.

However, in the UK, a 2018 survey of public contributors to National Institute of Health and Social Care Research (NIHR) funded research showed that the majority of people who get involved in research are over 50 years old, female and White [8]. The same survey in 2021 showed little change, with survey respondents predominantly female (57%), 61 years of age and over, White British (91%) and heterosexual [9]. People from lower socioeconomic groups and those experiencing racial inequalities feel less confident to be treated with dignity and respect in research [10]. Many reports have called for increased diversity and inclusion in PPIE [1]; initiatives and frameworks to address greater inclusion in PPIE are emerging eg. NIHR Race Equality Framework for Public Involvement [11], NIHR INCLUDE project [12], and the UK Public Involvement Standards (Inclusive Opportunities) [13].

An important aspect of demonstrating the effectiveness of any initiatives to increase inclusion of wider individuals and groups in PPIE is the need to collect and analyse data related to demographics of those taking part. However, at least in the UK, there is no systematic way or requirement to do so nationally: the NIHR surveys mentioned above are not (to the authors’ knowledge) conducted yearly or with any other regularity. The authors are aware of individual programmes and projects that do collect such data (for example, NIHR Biomedical Research Centres located elsewhere in the UK), but these approaches are not standardised nor widely published. Therefore, no routine data or comparator baseline exists against which to assess the diversity of PPIE. Much has been published on the complexity of how PPIE operates (from light touch input to intensive co-production), how its impact is judged, and the sensitive and power-laden contexts in which it operates, often working with those who are marginalised or feeling unwell [for example, 14, 15]. But if we don’t understand who is getting involved, there is an important part of the picture missing: meaningful public involvement is a matter of who gets involved, and how.

This paper reports on work carried out by Vocal [16], which aims to connect people and health research for everyone’s benefit. Vocal is a centre of excellence in PPIE in the UK, based in Greater Manchester (GM) in the North West of England, UK. GM has a population of 2,770,000 and is landlocked spanning 492.7 square miles (1276 km^2^). It includes ten Boroughs (metropolitan areas): a mix of high density urban areas, suburbs, semi-rural and rural locations, but overwhelmingly the land use in the county is urban. Since 2015, GM has adopted a devolved health and social care system enabling the region more power and control over budgets from central government, including in relation to health and social care.

Vocal is hosted by Manchester University NHS Foundation Trust (MFT) in partnership with the University of Manchester, and receives funding from the NIHR, the Wellcome Trust and others. It adopts a strategic and collaborative approach to PPIE across the Greater Manchester conurbation [17]. This is achieved by working as part of the NIHR Manchester Biomedical Research Centre (MBRC) [18] and NIHR Manchester Clinical Research Facilities (MCRF) [19], the North West Research Design Service, Wellcome funded research groups and other local research infrastructure.

At the time of the work reported here, Vocal’s programme mainly supports PPIE input to experimental medicine and translational research into cancer (prevention early detection, radiotherapy, precision medicine), hearing health, respiratory medicine, musculoskeletal and dermatology conditions (as part of the MBRC); maternal and fetal health and antimicrobial resistance, mainly in adult populations. Vocal’s activities include:

- Supporting lived experience input to individual research studies and programmes of research, through Research Advisory Groups and Networks. Activities included (but were not limited to): meetings to help develop grant applications, supporting public co-applicants, facilitated discussions about the accessibility and relevance of research methods, preferences on consent models, input to study recruitment strategies, comments on patient information sheets.
- Training, advising and supporting researchers, research staff and public contributors
- Co-creating (with researchers, public contributors and creative practitioners) large scale engagement campaigns focused on raising awareness of research, and how to have a say in research
- Strategic input to research infrastructure in GM, including through public contributor and Vocal staff membership of operational and strategic research committees.
- Supporting an ‘engaged research culture’.

Vocal recruits public contributors through a range of methods, including through clinical contacts, social media, relationships developed with patients groups, community, voluntary, civic and creative sector organisations, community centres, social change organisations working with marginalised communities.

The aim of the work presented in this paper was to capture and analyse the characteristics of who does and doesn’t take part in Vocal PPIE activities. In doing so, this contributes to a wider aim and motivation to become more inclusive in PPIE, by understanding who is and isn’t included in PPIE and targeting, through strategic initiatives and specific activities, those who remain excluded. Ultimately, the ambition is to foster more inclusive research. In this paper, we explore the feasibility of methods to capture demographic characteristics of people who are engaged and get involved in a vibrant early phase translational clinical research ecosystem in the North West of England.

## Methods

### Designing a questionnaire for recording demographic data

The NIHR MBRC and MCRF Health Inequalities Steering Group (HISG [20]) works to collectively address health inequalities in the research that is designed, delivered and communicated by the respective organisations. The group exerts strategic influence and conducts activities across GM research infrastructure, in order to promote inclusive research. Co-chaired by the Vocal Director and a Professor of Public Health and Epidemiology, the group includes public contributors, research support staff, researchers from different disciplines, Equality Diversity and Inclusion (EDI) personnel and PPIE practitioners. In response to the issue of lack of routinely collected demographic data related to PPIE and participation in experimental medicine research, Vocal worked with the HISG to design a questionnaire to capture the demographic characteristics of those who participate, are engaged and actively involved in clinical research. Here we report on the process for PPIE. The process for collecting data on participation will be reported elsewhere.

**Fig. 1:**
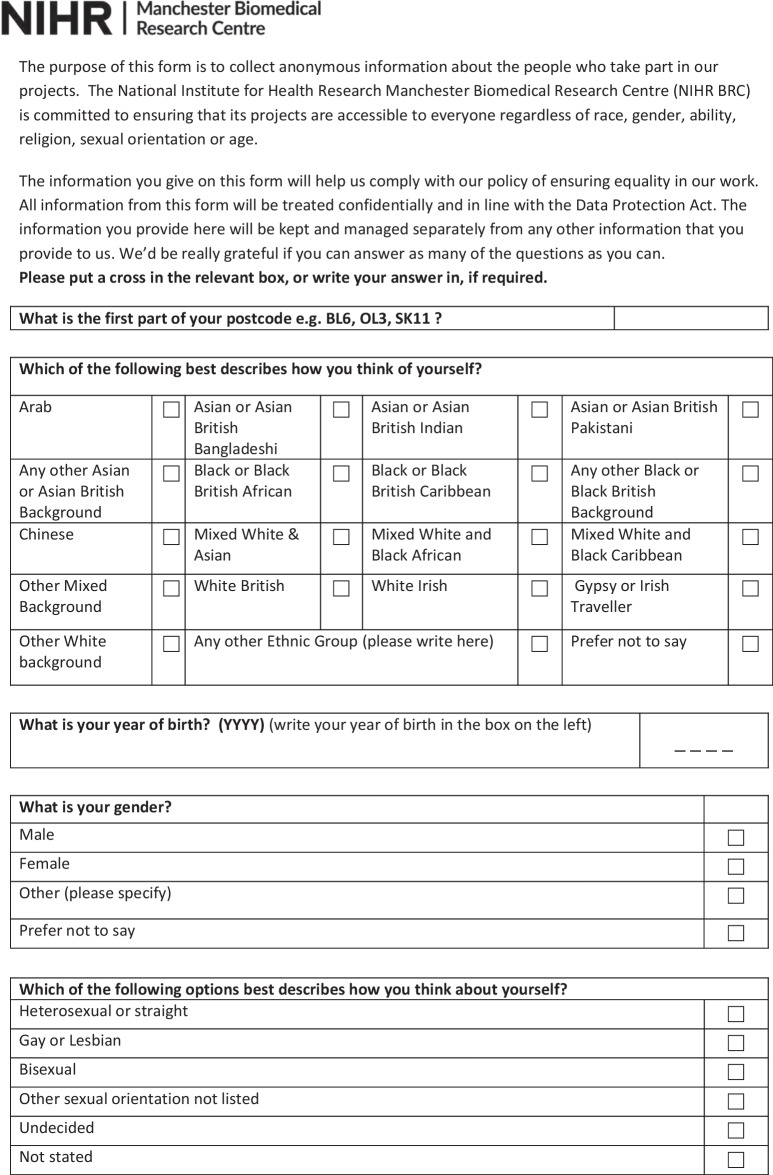

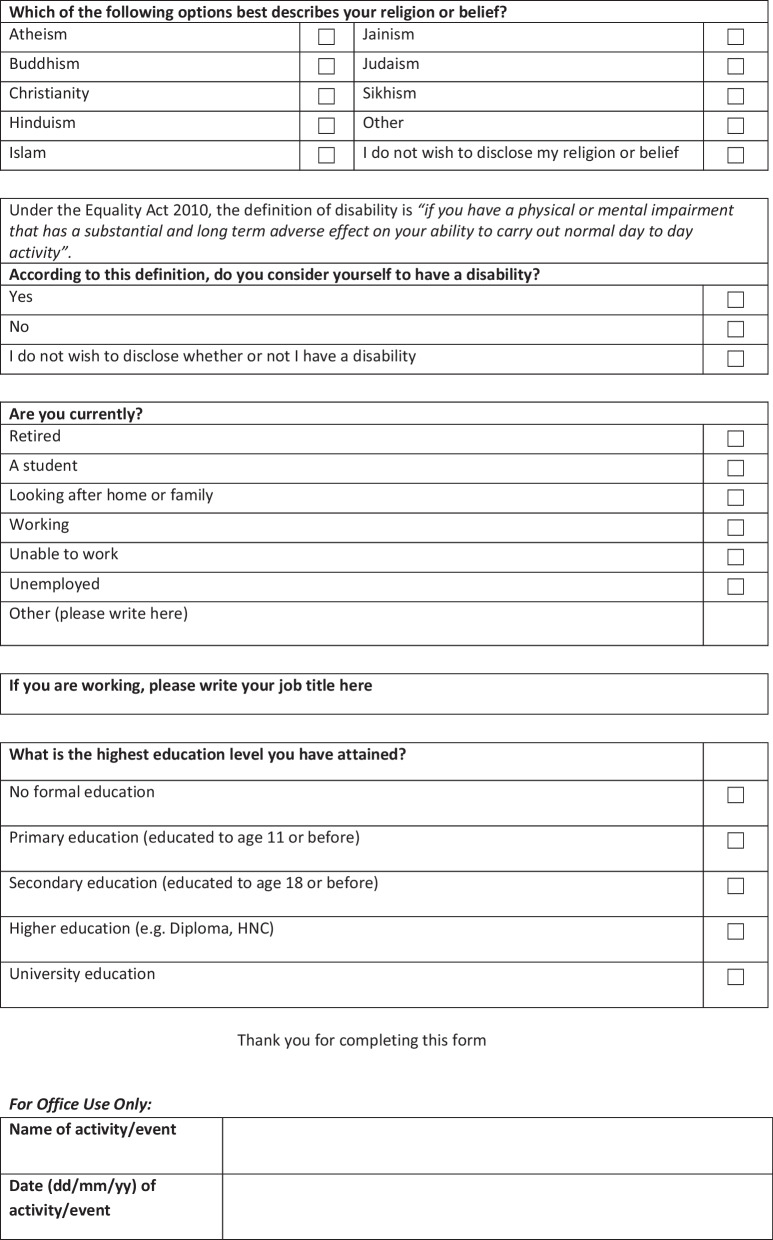
Demographic data questionnaire

The form was designed and the decision on which fields to capture was informed by existing good practice and advice received from the EDI team within MFT, public health monitoring practices (including the use of multiple indices of deprivation indicators), the Data Protection Act and the Equality and Human Rights Commission. The form was reviewed for accessibility by the HISG (including public contributor members of the HISG) which included discussions on what fields to include, and which ones to remove. For example, a field relating to caring responsibilities was removed.The questionnaire used for capturing demographic data for PPIE is shown in Fig. 1. The questionnaire did not capture identifiable information (ie. the name of the person completing the questionnaire was not recorded) and used tick boxes for recording other information. Fields in the form relate to protected characteristics as defined by the Equalities Act 2010 and include additional fields as proxies for socioeconomic status, including employment status, level of education and postcode. Postcodes in the UK are alphanumeric codes designating an area with several addresses or a single major postal delivery point. The first part of the alphanumeric code (called the outcode) has between 2 and 4 characters and designates a postal code district. The second alphanumeric part of the code is the incode, which contains 3 characters. The incode designates the postal code sector and delivery point and often relates to groups of around 15 addresses. In the UK, postcode and geographical location can be linked to lower super output areas (LSOAs)—a Census area of appropriate 1500 people and to the UK index of multiple deprivation [21].

The questionnaire preamble contains details of why demographic information is being requested and was drafted by members of the HISG.

In consultation with the Information Governance team of MFT (host organisation for Vocal and the MBRC and MCRF), and The Christie NHS Trust (partner site for the MCRF), the questionnaire was approved for use by the Information Governance Oversight Boards of each NHS Trust. Following feedback and instruction from Information Governance Oversight Boards during the initial development of the form, the incode was removed from capture from the questionnaire. This was deemed to be necessary by the Information Governance Oversight Board so as to avoid the collection of too much personal, identifiable data. The questionnaire further underwent a Data Protection Impact Assessment (DPIA) and its continued use is under regular and constant review by MFT’s Information Governance Team, with regular follow up DPIAs.

Having undergone thorough Information Governance review, it was not deemed necessary for the questionnaire to be submitted for ethical review. Further, the purpose of the questionnaire was to evaluate service provision (the ‘service’ being PPIE) rather than as a way of generating original research. As indicated by the Health Research Authority in the UK, PPIE activities are not considered research and therefore not subject to ethics review [22].

### Capturing demographic data related to PPIE

The form developed above was introduced to Vocal’s routine PPIE practice, to:

- Explore the feasibility of collecting demographic data from those taking part in PPIE activities
- Describe the demographic characteristics of people involved in Vocal’s activities, during a period between 2018 and 2022 (a ‘snapshot’).

The demographic questionnaire was distributed at all Vocal-led PPIE events held between December 2018 and end of March 2022. Completion of the questionnaire was voluntary, and people were not obliged to answer any or every question. People were asked whether they wanted to complete the questionnaire.

Data received were held in the Vocal Client Relationship Management (CRM) system. This is a contacts management database for PPIE set up within Vocal in line with Data Protection and Information Governance requirements, and using a bespoke platform (called GoodCRM). Data were stored in accordance with MFT’s General Data Protection Regulations (GDPR).

The form was initially distributed in paper form during Vocal PPIE activities, for example meetings of Vocal’s Research Advisory Groups [23], engagement activities in the time period (focused on the research areas of the MBRC eg. Hearing Health Now [24]) and to public contributor members of research governance committees. The form was moved online in May 2021, using a secure survey platform (Qualtrics) hosted by the University of Manchester. Electronically, the form was filled out by public contributors independently in advance of or following PPIE online and in person activities, when follow up contact was made (for example when processing payments for PPIE and/or as part of evaluation activities). Where needed, Vocal staff provided support to public contributors if they had questions about the purpose of collecting data, needed further explanation and advice, or were unsure which categories to fill in.

### Statistical analysis and comparator data

The results are of the questionnaire are summarised by numbers and percentages according to category (Table 1). To assess whether this sample of people are different from the GM population or national PPIE contributors two datasets were used as comparator datasets and also presented as number and percentages (Table 1).

**Table 1:** Demographic characteristics of people taking part in Vocal PPIE activities, cumulative data December 2018 to March 2022

Ethnicity	Vocal public contributors (n = 329)		NIHR public contributors 2018 (n = 631) 2021 (n = 819)	GM Census Data (2011)
	N°	%	%	%
White British	178	54.10	No data	79.8
White Irish	5	1.51		1.29
White Gypsy Roma Traveller	0	0		0.67
Other White Background	4	1.21		2.62
White (total)	187	56.84	77 (in 2018) 91.5 (in 2021)	84.38
Asian or Asian British Indian	16	4.86	No data	1.99
Asian or Asian British Pakistani	53	16.11		4.85
Asian or Asian British Bangladeshi	5	1.52		1.27
Asian Chinese	9	2.74		0.97
Any other Asian or Asian British Background	3	0.91		1.06
Asian or Asian British (total)	86	18.54	3 (in 2018) 2.2 (in 2021)	10.14
Black or Black British Caribbean	14	4.25	No data	0.66
Black or Black British African	10	3.04		1.67
Any other Black or Black English Background	1	0.30		0.43
Black or Black British (total)	25	7.60	2 (in 2018) 2.9 (in 2021)	2.26
Mixed White and Black Caribbean	0	0	No data	0.86
Mixed White and Black African	2	0.61		0.37
Mixed White and Asian	1	0.30		0.58
Other Mixed background	3	0.91		0.44
Mixed (total)	6	1.82	2.6 (in 2021)	1.02
Arab	13	3.95		0.56
Any other ethnic group	5	1.51		0.46
Prefer not to say	7	2.13		
Sex				
Male	120	36.47	40.7 (in 2021)	49.44
Female	207	62.92	56.7 (in 2021)	50.56
Religion				
Christian	101	30.70	No data	61.79
Buddhist	7	2.13		0.36
Hindu	4	1.22		0.88
Jewish	8	2.43		0.93
Muslim	84	25.53		8.68
Sikh	0	0		0.20
Other religion	23	6.99		0.28
No religion	61	18.54		20.77
Not stated	21	6.38		6.12
Sexuality				
Heterosexual	287	87.23	86.4 (in 2021)	
Gay or Lesbian	6	1.82	1.3 (in 2021)	
Bisexual	11	3.34	3.8 (in 2021)	
Other	1	0.30	2.3 (in 2021)	
Undecided	2	0.61		
Not stated	21	6.38	5.9 (in 2021)	
Do you consider yourself to have a disability?				
Yes	87	26.44	‘A lot’: 15.6 ‘A little’: 31.9	
No	209	63.53	‘Not at all’: 50.6	
Prefer not to say	31	9.42	1.9	
Are you currently…				
Retired	61	18.54	66.48 aged 61 or over (in 2021)	
Student	72	21.88		
Looking after home or family	23	6.99		
Working	111	33.74		
Unable to work	21	6.38		
Unemployed	14	4.25		3.5
Other	23	6.99		
What is your highest level of education?				
No formal education	8	2.43		
Primary education	3	0.91		
Secondary education	91	27.66		
Higher education	75	22.80		
University education	141	42.86		

Data are compared against national surveys of public contributors carried out by the NIHR in England in 2018 [7] and 2021 [8], and against Census data relating to Greater Manchester from 2011

GM population data were sourced from the 2011 Census [25]. At the time of writing, the 2021 Census results have yet to be published but would provide more up to date population data comparisons.

Comparator data about the demographics of PPIE contributors, nationally, were sourced from two surveys—one in 2018–2019 and one in 2020–2021—carried out by NIHR in the UK [8, 9]. The first survey ran from December 2018 to January 2019, receiving 809 responses with a 72% completion rate. Headline data indicate that young people and minority ethnic communities were under-represented in NIHR’s public involvement at the time: younger age groups represented 2% of survey respondents (under 25) and 14% of respondents (aged 26–49). 3% of respondents were from Asian ethnic groups, and 3% from Black ethnic groups. Respondents were generally older, predominantly white and female (58% women, 35% men, 7%). 74% identified themselves either as patients, service users or members of the public with lived experience.

The second survey ran from July 2021 and consisted of 24 questions. These were a mix of questions which asked respondents to select answers from options given to them, and questions that enabled respondents to answer in their own words (free text). People were free to answer as many of the questions as they wanted. This meant not all respondents answered every question. There were 819 responses to the survey. Survey respondents were predominantly female (57%), 61 years of age and over, white and heterosexual. 47% of respondents (268 of 565) stated they had physical or mental health conditions, disabilities or impairments (most commonly mobility issues) that limited their ability to carry out certain tasks. Both NIHR surveys reported headline data via their websites. A more detailed report of the 2021 NIHR survey was made available by NIHR and provided on request to the authors of this paper. Data reported in Table 1 related to the 2021 NIHR survey are sourced from this more detailed report.

It should be noted that the NIHR surveys were not focused on demographics of public contributors and explored wider experiences of PPIE. This means that information related to the characteristics of public contributors were not always exactly comparable with our data. For example, NIHR surveys reported some ethnicity as related to being Black or Asian, without further granularity (eg. Black British African, Asian British Pakistani). Our survey categorised disability as according to the definition from the Equalities Act 2010, whereas in the NIHR 2021 survey respondents self-reported mental or physical health condition, disability or impairment that limited their ability to carry out tasks ‘a lot’, ‘a little’ or ‘not at all’. Where possible, data from our work have been compared against the most relevant comparator data set (Table 1). We reference comparator data from both NIHR surveys, as they both cover the time period covered by our questionnaire.

## Results

Between December 2018 and the end of March 2022, a total of 329 forms were returned. Table 1 summarises the data received and compares against the demographics of local GM population (Census data 2011) [25] and data from the 2018 and 2021 NIHR national surveys of public involvement [8, 9]. Our findings indicate that assessing the demographic characteristics of people taking part in PPIE activities is possible, and that the people taking part in Vocal’s PPIE activities are more ethnically diverse, and represent a greater diversity in age range, than national indicators. Data also show good representation across other characteristics (including employment status). We attribute the diversity of contributors to Vocal’s PPIE activities as a result of Vocal’s strategic priority (2017–2022) [26] to increase inclusion in PPIE.

### Return rate and completion of questionnaire

As of end of March 2022, 329 of Vocal’s public contributors had returned the demographic monitoring questionnaire, out of a total of 935 public contributors associated with Vocal at end of March 2022, so a return rate of 29.3%.

Most people filling in the form completed all the fields, though there were a few forms that omitted some fields. The highest error rate was in the field capturing postcode, with 53 answers being illegible, not corresponding to a postcode, or left blank. We interpret this to mean that the questionnaire was generally considered accessible, especially the answers that required ticking a box, with errors introduced in the free text field capturing postcode. Vocal staff were able to support people to fill in the questionnaires, and this was occasionally needed. Where there might be sensitivity about answering some questions, ‘Prefer not to say’ options were sometimes ticked, suggesting that this field was appreciated by respondents.

### Ethnicity

18.54% of people taking part in Vocal’s PPIE activities self-identified as Asian (including Asian or Asian British Indian, Asian or Asian British Pakistani, Asian or Asian British Bangladeshi, Asian Chinese and any other Asian or Asian British Background). This compares to 3% (in 2018) and 2.2% (in 2021) of public contributor respondents to NIHR surveys identifying as Asian (no sub-categorisation of Asian ethnicities). This indicates that public contributors taking part in Vocal PPIE activities include significantly more people of Asian heritage than national indicators. 10.14% of the GM population identify as Asian in 2011, indicating that Vocal is working with greater numbers of Asian contributors when compared to population demographics. However, most of Vocal’s public contributors are from the GM Borough of the City of Manchester, which includes 17.1% of the population of Asian heritage—which is in line with Vocal’s Asian public contributor demographics.

7.60% of people taking part in Vocal’s PPIE activities self-identified as Black (including Black or Black British Caribbean, Black or Black British African and any other Black or Black British background). This compares to 2% (in 2018) and 2.9% (in 2021) of public contributor respondents to NIHR surveys identifying as Black (no sub-categorisation of Black ethnicities). This indicates that public contributors taking part in Vocal PPIE activities include significantly more people of African or Caribbean heritage than national indicators. 2.26% of the GM population identify as Black in 2011, indicating that Vocal is working with greater numbers of Black contributors when compared to population demographics. However, most of Vocal’s public contributors are from the GM Borough of the City of Manchester, which includes 8.60% of the population of African or Caribbean heritage—which is in line with Vocal’s African and Caribbean public contributor demographics.

The percentage of people of mixed heritage taking part in Vocal’s PPIE activities (1.82%) is similar to national indicators of PPIE public contributors (2.6% in 2021 NIHR survey) and the GM population (1.02%).

### Religion

Our data show that the majority of people taking part in Vocal PPIE activities are Christian (30.70%) or Muslim (25.53%). No comparator data were available from NIHR surveys of public contributors. However, when comparing with GM population data, we see greater numbers of public contributors are of Muslim, Jewish, Buddhist, Hindu and other faiths than are represented in the GM population. We include significantly fewer people of Christian faiths in PPIE activities, when compared to proportions of people in the GM population.

### Sex and gender

Most people taking part in Vocal’s PPIE activities are women (62.92%) compared to 36.47% men. This represents a less equal balance than national indicators of PPIE: the NIHR survey in 2021 includes 56.7% female respondents and 40.7% male respondents. In GM, there are slightly more women (50.56%) than men (49.44%).

It should be noted that Vocal’s process for collecting information related to their sex and gender has been updated since the questionnaire reported here. Now, in addition to asking about sex (male/female) we ask if the gender the respondent identifies with is the same as their sex registered at birth, providing an option for respondents to enter their gender identity (in line with the approach used in the 2021 Census).

### Sexuality

The proportions of people taking part in Vocal’s PPIE activities identifying themselves as heterosexual, gay or lesbian, and bisexual is similar to national NIHR indicators related to public contributors’ sexuality. 87.23% of Vocal public contributors identify as heterosexual compared to 86.4% in the NIHR survey conducted in 2021.

### Working status and level of education

Our data indicate a good mix of public contributors from across different working status and levels of education. Working status and level of education were included in our monitoring, as proxy indicators of socioeconomic status, and were considered important especially in the absence of being able to capture full postcode (which would allow more granular analysis of indices of deprivation). Comparator data for working status and level of education are not available, but it is widely considered that public contributors tend to come from backgrounds with high levels of education. This is borne out by our findings, with the majority—141 respondents (42.86%)—reporting a University education. However, we were encouraged to see that 91 respondents (27.66%) reported a secondary education as their highest level of education, suggesting that we are reaching considerable numbers of people without further or higher education. Likewise, the majority of people who take part in Vocal activities are working (111 or 33.74%), suggesting that we are not reliant on those who are retired and/or in a privileged working status to take part in PPIE activities.

### Age

**Fig. 2:**
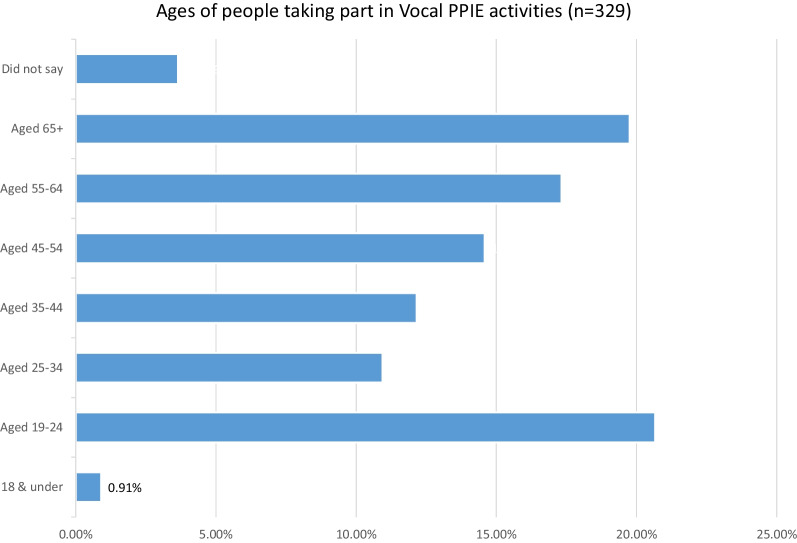
Relative percentages of people taking part in Vocal PPIE activities, by age

Table 2 details public contributors to Vocal PPIE activities, by year of birth. The questionnaire asks for year of, birth rather than self-reported age, so that when age data are interrogated at later dates than the form completion date, they accurately report continuous age. We find that public contributors to Vocal PPIE activities are from all ages, with a relatively even spread. The highest numbers of public contributors—68 or 20.67%—have their year of birth between 1998 and 2003 (aged 19–24 in 2018) followed by those with a year of birth before 1958 (aged 65 + in 2018) at 65 or 19.67%. However, all other ages are well represented (Fig. 2). When compared with national data from NIHR surveys, our results show a more diverse representation of age in PPIE activities: the majority of public contributors to NIHR research are over 61 years of age. NIHR respondents were most likely to be in the 71 to 80 year old age group (31.4%); 61 to 70 was the second most represented age group (30.1%); and the least represented age brackets were under 18 years old (0.4%), 19 to 30 year olds (1.9%), and 31 to 40 year olds (3.6%). Although the age categories do not match directly across Vocal age data and age data obtained from NIHR, we are confident in concluding that Vocal PPIE activities include people from a wider range of ages than national indicators. The spread of ages may be attributable to the activity of Vocal’s Young Person’s Advisory Group (called Voice Up) during the period of this work. Voice Up is a group of young people from diverse backgrounds who act as advisors to research and Vocal’s work. While some Voice Up members are students, Vocal’s practice is to generally avoid recruiting University students to PPIE activities (although this is sometimes an easy route to recruitment) so as to mitigate over-representation of privileged and/or educated status (cf. Working Status section). Most people living in Greater Manchester are adults aged 20–24 and 25–29 [27].

**Table 2:** Ages of people taking part in Vocal PPIE activities

Age of public contributor	Number	Percentage
Year of birth after 2003 (18 and under in 2018)	3	0.91
Year of birth 1998–2003 (aged 19–24 in 2018)	68	20.67
Year of birth 1988–1997 (aged 25–34 in 2018)	36	10.94
Year of birth 1978–1987 (aged 35–44 in 2018)	40	12.16
Year of birth 1968–1977 (aged 45–54 in 2018)	48	14.59
Year of birth 1958–1967 (aged 55–64 in 2018)	57	17.32
Year of birth before 1958 (aged 65 + in 2018)	65	19.76
Did not say	12	3.65

### Geographical spread

**Fig. 3:**
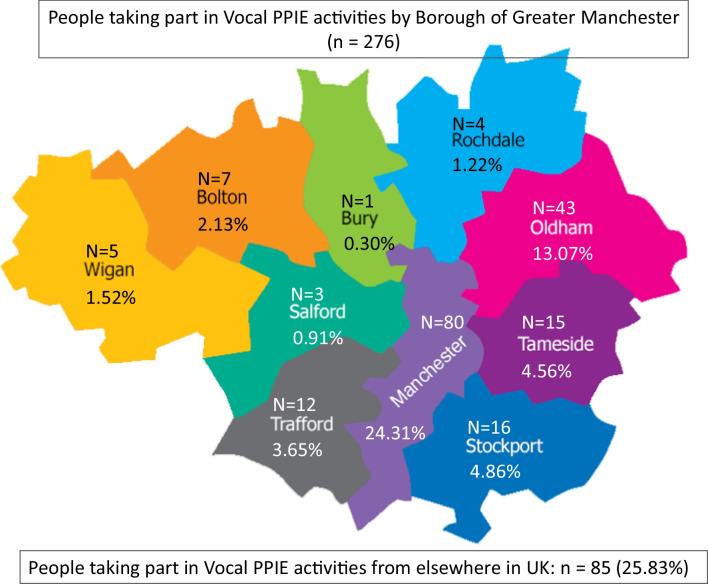
People taking part in Vocal PPIE activities by Borough of Greater Manchester

Those taking part in Vocal’s PPIE activities come from Greater Manchester and beyond, from different areas of the UK. The majority of Vocal’s public contributors (58.05%) live in GM, with 25.83% living outside of GM. Figure 3 maps public contributors against Boroughs of GM, showing the greatest number (80 or 24.31% of total contributors) from the Borough of the City of Manchester. The Borough of Oldham shows the second highest numbers of people taking part in Vocal PPIE activities. Data may reflect proximity of people to Vocal’s geographical base and locality-based initiatives, as well as ability to travel to events.

Further analysis of demographic characteristics mapped onto individual GM Boroughs, which would allow further granularity in terms of who Vocal is reaching, Borough by Borough, is possible though is not presented here. Understanding reach in terms of geographical areas outside of GM would also be possible, including in rural areas: GM secondary and tertiary health services serve a wide catchment area, including rural locations.

## Discussion

The findings presented here indicate that assessing the demographic characteristics of people taking part in PPIE activities is possible, and that the people taking part in Vocal’s PPIE activities are more ethnically diverse than national PPIE indicators, represent wider age ranges when compared to national PPIE indicators, and include good representation across other characteristics. This is probably due to Vocal’s strategic priority (2017–2022) [26] to increase inclusion in PPIE.

### Feasibility of capturing demographic data related to PPIE

The introduction of a demographic monitoring process for PPIE is feasible and provides useful data to understand who we are (and aren’t) reaching in public involvement in research. In order to have a fully rounded understanding of PPIE, there is a need to understand both the qualitative and the quantitative dimensions of PPIE. The approach and data described here are useful as part of a wider programme of PPIE that is also informed by qualitative approaches, and by partnership working: all of Vocal’s work is carried out collaboratively with public contributors.

However, in the absence of any standard or mandated practice of capturing information on the characteristics of people who get involved in research, the process described here provides an overview of an approach, and may be transferable to other similar PPIE contexts. Learning from and evolving the practice of capturing demographic data during this approach from December 2018 to March 2022 has informed Vocal’s current practice of understanding who is (and isn’t) involved in PPIE activities The existence of baseline data against which to compare and benchmark the diversity of PPIE activities and progress is sparse, and external comparators might be limited by differences in questions and reach of other comparators. Nevertheless, the ‘snapshot’ of data reported here can serve as useful information for ongoing work and the data that is now collected routinely provides rich and meaningful insights into Vocal’s strategic direction and programme of PPIE work. For example, in responding to the data presented here related to gender balance, and sexuality, the Vocal team has focused on targeting more men to be involved in some PPIE activities; and has developed partnerships with organisations in the LGBTQIA + space.

### A more diverse public contributor community?

Whilst this was not a comprehensive analysis, the results indicate that Vocal is reaching a greater diversity of people, compared to national indicators from NIHR, in PPIE activities supporting the GM clinical translational research ecosystem (and MBRC research more specifically). In particular, data indicate that greater numbers of people with experience of racial inequalities contribute to Vocal PPIE, when compared to available national data (NIHR surveys of public contributors 2018 and 2021). The percentages of people with experience of racial inequalities included in Vocal PPIE activities mirrors the demographics of the GM population, as indicated with reference to the 2011 GM Census. We await the results of the 2021 Census. When conducting subset analyses, we note that the percentages of people of Asian, African and Caribbean heritage most closely approximate the populations in the Borough of the City of Manchester, where most of Vocal’s public contributors come from (Fig. 3). We attribute the relatively high levels of contribution from people of Asian, African and Caribbean heritage, to our strategic focus on increasing inclusion in PPIE within these populations over the last 5 years. This includes initiatives such as the community sandpit [28] and the formation of BRAG—Vocal’s Black Asian and Minority Ethnic Research Advisory Group [29]. The Community sandpit, held in 2018, was a joint venture between the Greater Manchester Black and Minority Ethnic Network, Vocal and brought together community organisations, artists and researchers and designed to promote new conversations between health researchers and people from diverse and marginalised groups. It aimed to shift the power balance, and to encourage and fund innovations in PPIE suggested by community members. BRAG emerged from the community sandpit. Bringing together 4–6 community leaders previously involved in the sandpit, the Group meets monthly, facilitated by Vocal staff. Since 2019, it has:

- Influenced 14 research projects
- Co-developed 3 grant applications
- Produced Top Tips for researchers wanting to work with BRAG
- Co-created Inclusive Research training [30]
- Received leadership training and learning opportunities about clinical research
- Reached over 5000 people to raise awareness of research and how to have a say in research, through community engagement
- Produced videos [eg. 31], blogs and personal reflections about working in partnership as part of health research
- Influenced the strategic focus of the NIHR Manchester Biomedical Research Centre’s renewal in 2022, towards inclusive research and race equity

In moving forward, BRAG members are part of the NIHR Manchester Biomedical Research Centre’s Governance functions and members of BRAG have co-led the NIHR Race Equality Framework for Public Involvement pilot in GM, across the nine NIHR infrastructure organisations in GM, resulting in 14 actions for change to be implemented towards greater race equality in research.

Our data also indicate that we are working with people across a wider range of ages, when compared to national indicators. This may be due, in part, to the success of Vocal’s Voice Up group, a research advisory group of young adults aged 16–24 who input into MCRF and MBRC programmes and projects, and to engagement initiatives during the time period reported here, that work with younger age groups [32, 33]. For example, The AudioLab [33] is a continuing partnership between Vocal, Reform Radio—a digital radio station in GM and social change organisations—that co-creates engagement with science with young people at relative socioeconomic, health or educational disadvantage. During the time of the work reported here, the global Planet DIVOC-91 project [34], engaged young people aged 16–24 with COVID research and supported their voices in influencing COVID research and policy.

The gender balance of Vocal’s work is skewed towards greater involvement of women. Vocal’s data is comparable to national data related to gender and PPIE and will inform our approach in going forwards to involve more men.

The percentage of those with a disability who get involved in Vocal’s PPIE activities is lower than national indicators. This may be due to ambiguities in the definitions: Vocal’s questionnaire uses the definition of the Equalities Act 2010, whereas the NIHR survey used self-reporting indicators of physical or mental health conditions, disabilities or impairments (most commonly mobility issues) that limit ability to carry out certain tasks. Whilst all of Vocal’s work is focused on PPIE, the organisation focuses on establishing and maintaining relationships with large numbers of contributors and organisations from a variety of different communities (of geography, characteristics and/or identity) as well as patients, carers and people from all walks of life. This means that activities are not limited to including people with lived experience of a particular health condition, though these groups are a pivotal part of Vocal’s work as represented through a wide range of condition specific research advisory groups and networks [23].

The distributions of religion, level of education and working status show a good range and that Vocal’s public contributors come from across the UK and from across all Boroughs of GM. The largest number of public contributors—from the Borough of the City of Manchester—may be due to the fact that Vocal is physically located within the City of Manchester, and the Borough also contains the greatest concentration of research active institutions (for example, MFT and the University of Manchester). The Borough of Oldham includes the second largest number of public contributors to Vocal’s work. This is attributable to Vocal’s strategic focus on working in this area during the time period reported here. Oldham is the Borough of Greater Manchester with the highest level of socioeconomic deprivation and it was decided to focus some of Vocal’s activities on this area during 2017–2022 to help address health inequalities. This included projects that raised awareness of health research, and how to have a say in health research, related to:

- Hearing Health (Hearing Health Now [24]).
- Radiotherapy Research (Radiotherapy & Me) [35]
- Research into musculoskeletal conditions (#MyMSKStory) [36]
- Mental Health (as partners in the Ideas Fund [37], which aimed to support community-led initiatives related to mental wellbeing)

### Limitations and next steps

This work reported in this paper adopts a ‘learn by doing’ approach. Throughout, the Vocal team have reflected, in collaboration with the HISG and wider research and public contributor communities, on the limitations of the process described in this paper which include:

- Return rate: a return rate of 29.3% for the survey questionnaire, whilst deemed to be decent, could be better. The return rate for comparator NIHR surveys is higher. It should be noted, however, that not all NIHR survey respondents answered all questions and that the large majority of Vocal questionnaire respondents answered all questions, with the highest error rate being in postcode completion. Much of this work was also carried out during the period of COVID restrictions, which may have had an effect on return rate. During this time, the survey was transitioned from a paper form completion, to an online form completion. Digital inequalities may prevent more marginalised public partners from completing the form.
- Duplication: there may have been an element of duplication within responses though this has not been systematically appraised. Where public contributors were involved in several Vocal activities during the time period of monitoring, they may have completed the questionnaire more than once. The standard practice was to complete the form ahead of PPIE activities, at which point, contributors were asked to indicate whether they had already completed it, and if they had, they were asked not to complete it again. In this way, duplication may have been minimised.
- Length of the form: the form requires responses to a large number of questions and this could be off putting for some. We worked with public contributor, public health and EDI expertise in the development of the form who collectively concluded that it would not accurately reflect the needs of both researchers, public engagement practitioners and public contributors if we removed any of the fields. Personal support (from Vocal staff) was provided for public partners to complete the form.
- Data collected: in initial DPIAs, it was recommended that the latter part of postcodes be removed, in order to minimise identification of respondents. This has presented limitations when analysing the data to understand the geographic and socioeconomic profile of public contributors, with a view to increasing their involvement. The first three digits of a postcode cover a large area which contain significant differences in indicators of deprivation—often from the highest decile of the Index of Multiple Deprivation (IMD) to its lowest decile. LSOAs provide better granularity (see Methods). By only including the first three digits of the postcode, it was difficult to more fully analyse who was engaged and involved in Vocal’s activities according to indicators of deprivation
- Comparator data: benchmarking Vocal’s data against existing datasets, was limited due to the relative paucity of comparator data. NIHR public contributor data provide the best comparison, together with GM population data. Vocal’s data to date, provide baseline data for the organisation in going forwards.

To address the limitations above, we have worked closely with Information Governance teams across our host NHS organisations. From March 2022, following a further DPIA, it has been agreed that Vocal can:

- Collect full postcode details of public contributors (in addition to the data already collected)
- Link demographic data to the personal record of public contributors within Vocal’s secure CRM system. This has been enabled through the use of a different CRM platform (Zoho), which provides greater functionality and which is still subject to and compliant with all GDPR and Information Governance requirements.

**Fig. 4:**
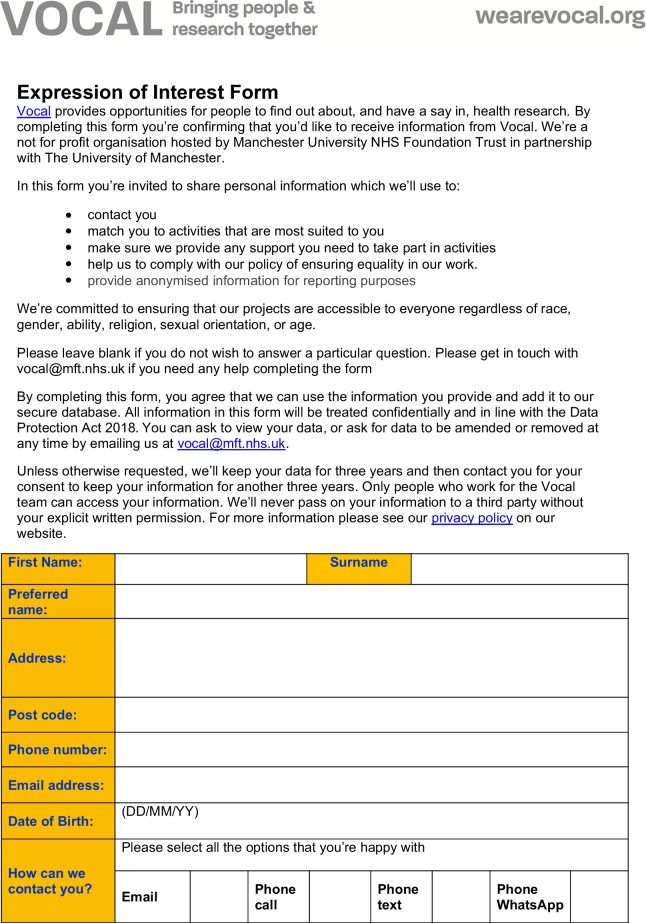

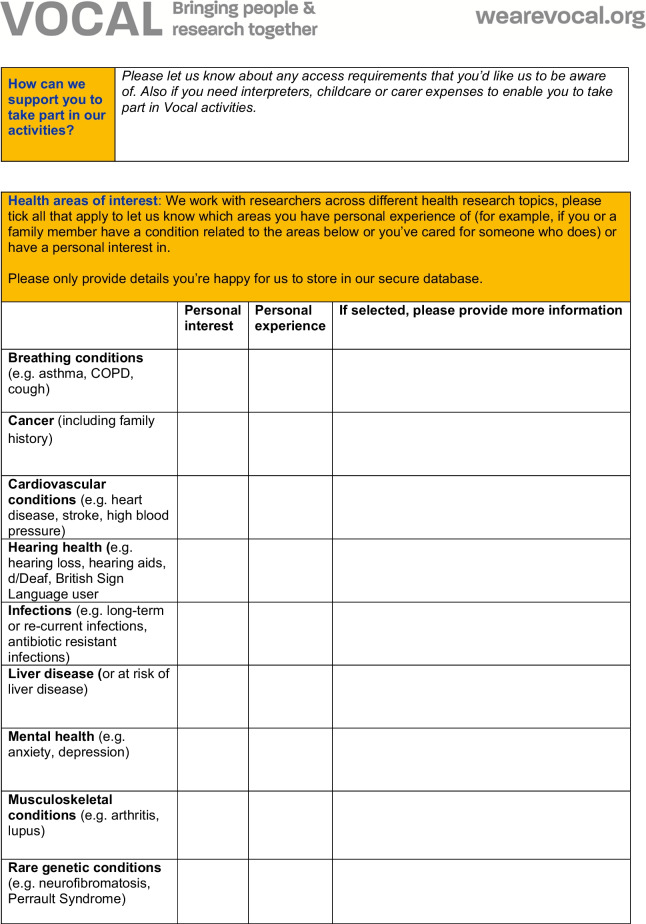

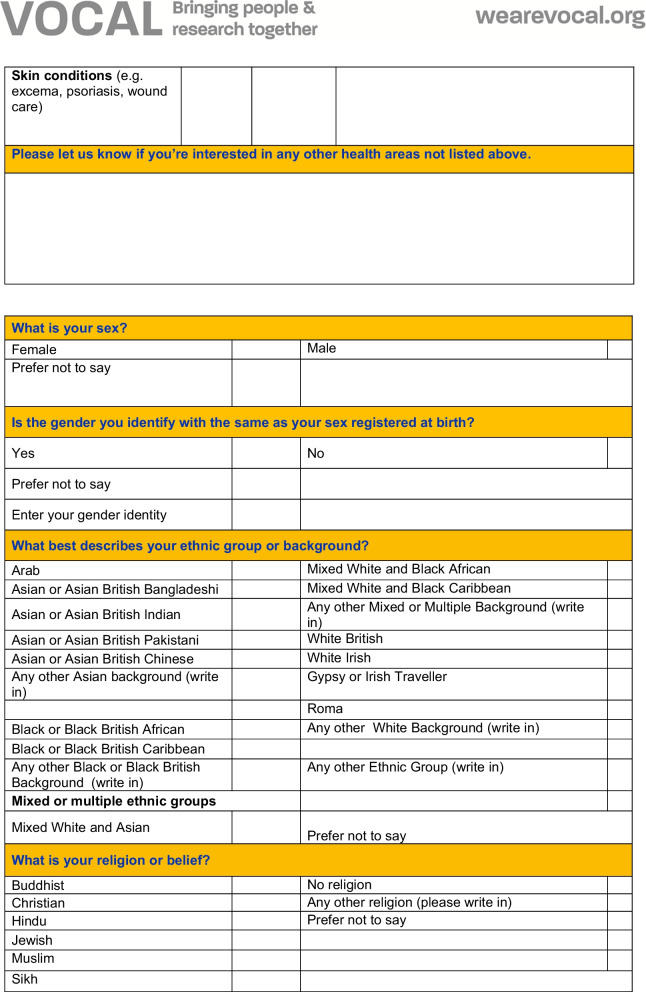

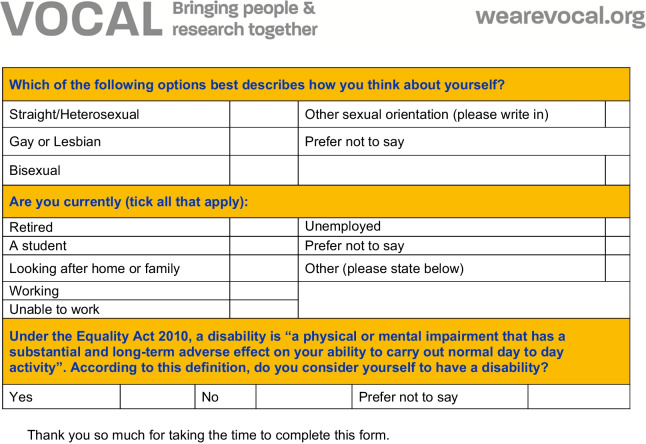
Updated questionnaire for completion by public partners

For clarity, no data from public contributor’s personal health records is kept on Vocal’s CRM system, and providing demographic data is still voluntary. But, by having a much more systematic approach to the logistics of engaging with public contributors, we are better able to target opportunities for involvement to specific communities (of geography, characteristics or identities), avoid duplication, avoid public contributor ‘fatigue’ through being asked to contribute to too many things, to systematically feedback the impact of involvement and maintain up to date records and contacts. An updated questionnaire is included for reference in Fig. 4, which also captures information about which health areas public partners are interested to collaborate on (without asking for specific health information). The questionnaire is completed upon first contact between public partners and Vocal and translation of the form is possible. Vocal staff support public partners to complete the questionnaire (for example, by taking the time to further explain the purpose of the form and/or provide clarity on which characteristics apply to them).

Data related to the demographics of public partners involved in Vocal’s work are reviewed quarterly by Vocal senior management, and by governance functions of the MBRC and MCRF. With the amendments made as above, it is also possible to drill down on diversity data in PPIE as it relates to, for example, research area (eg. musculoskeletal research carried out in the MBRC). By also openly publishing headline data about the characteristics of the people it works with [38], Vocal aims to ensure transparency of its work.

Staff engagement has been essential to the process of capturing demographic data, including within Vocal staff and wider host organisations and partners. We have shared our process informally with other local organisations, who are adopting this way of working. Since the submission of this paper, a number of national (UK) initiatives are now considering whether and how to collect demographic data related to research involvement, engagement and participation. For example, the NIHR is considering how to capture diversity data related to participation and involvement in health and social care research, as is the Research Engagement Network Development programme (led by NHS England).

## Conclusion

Since 2017, Vocal has prioritised increasing the diversity of people who get engaged and involved in research, through developing a strategic approach and more inclusive methods of PPIE. Part of this strategic approach has been to develop methods for capturing, monitoring and reporting who Vocal is (and isn’t) reaching with its activities. The work presented here summarises the approach taken and data indicate that Vocal is taking steps to meet its ambitions to diversify PPIE, to ultimately produce more inclusive research that is relevant to, and benefits all of us.

## Data Availability

The datasets used and/or analysed during the current study are available from the corresponding author on reasonable request. All data generated or analysed during this study are included in this published article.

## Abbreviations

BRAG: Black Asian and Minority Ethnic Research Advisory Group
CRM: Client relationship management
DPIA: Data protection impact assessment
EDI: Equality, diversity and inclusion
GDPR: General data protection regulation
GM: Greater Manchester
HISG: Health Inequalities Steering Group
IMD: Index of multiple deprivation
LSOA: Lower super output area
MFT: Manchester University NHS Foundation Trust
MBRC: NIHR Manchester Biomedical Research Centre
MCRF: NIHR Manchester Clinical Research Facility
NIHR: National Institute of Health and Social Care Research
PPIE: Patient and public involvement and engagement

## References

[1] Staniszewska S, Denegri S, Matthews R, Minogue V (2018). Reviewing progress in public involvement in NIHR research: developing and implementing a new vision for the future. BMJ Open.

[2] HRA and INVOLVE Briefing note: Impact of public involvement on the ethical aspects of research. https://s3.eu-west-2.amazonaws.com/www.hra.nhs.uk/media/documents/impact-public-involvement-ethical-aspects-research-updated-2016.pdf. Accessed 15 November 2022.

[3] NIHR Briefing notes for research: public involvement in NHS, health and social care research. https://www.nihr.ac.uk/documents/briefing-notes-for-researchers-public-involvement-in-nhs-health-and-social-care-research/27371#Improving_the_quality_of_the_research. Accessed 15 November 2022.

[4] Staley K. Changing what researchers 'think and do': Is this how involvement impacts on research? Research for All 2017; 10.18546/RFA.01.1.13.

[5] Crocker (2018). Impact of patient and public involvement on enrolment and retention in clinical trials: systematic review and meta-analysis. BMJ.

[6] Marmot M, Health Equity in England: The Marmot Review 10 Years On. https://www.health.org.uk/publications/reports/the-marmot-review-10-years-on. Accessed 15 November 2022. 10.1136/bmj.m693 32094110

[7] Bower P, Grigoroglou C, Anselmi L (2020). Is health research undertaken where the burden of disease is greatest? Observational study of geographical inequalities in recruitment to research in England 2013–2018. BMC Med.

[8] Taking Stock: NIHR public involvement and engagement 2019. https://www.nihr.ac.uk/documents/taking-stock-nihr-public-involvement-and-engagement/20566#NIHR_public_contributors%E2%80%99_feedback_survey. Accessed 15 November 2022.

[9] NIHR Public Involvement Feedback Survey 2020–2021: The results. https://www.nihr.ac.uk/documents/nihr-public-involvement-feedback-survey-2020-2021-the-results/29751#:~:text=Respondents%20reported%20contributing%20to%20the,people%20contribute%20from%20different%20perspectives. Accessed 15 November 2022.

[10] Hunn A. Survey of the general public: attitudes towards health research 2017. https://s3.eu-west-2.amazonaws.com/www.hra.nhs.uk/media/documents/HRA_NIHR_general_public_omnibus_survey_2017_FINAL.pdf. Accessed 15 November 2022.

[11] NIHR Race Equality Framework 2022 https://www.nihr.ac.uk/documents/nihr-race-equality-framework/30388. Accessed 15 November 2022.

[12] Treweek S, Banister K, Bower P (2021). Developing the INCLUDE Ethnicity Framework—a tool to help trialists design trials that better reflect the communities they serve. Trials.

[13] UK Standards for Public Involvement. https://sites.google.com/nihr.ac.uk/pi-standards/home?pli=1. Accessed 15 November 2022.

[14] Ocloo J, Matthews R (2016). From tokenism to empowerment: progressing patient and public involvement in healthcare improvement. BMJ Qual Saf.

[15] Russell J, Fudge N, Greenhalgh T (2020). The impact of public involvement in health research: what are we measuring? Why are we measuring it? Should we stop measuring it?. Res Involv Engag.

[16] Vocal. https://wearevocal.org/. Accessed 15 November 2022

[17] Holmes (2019). Innovating public engagement and patient involvement through strategic collaboration and practice. Res Involv Engagem.

[18] Manchester Biomedical Research Centre. https://www.manchesterbrc.nihr.ac.uk/. Accessed 15 November 2022.

[19] Manchester Clinical Research Facility. https://manchestercrf.nihr.ac.uk/. Accessed 15 November 2022.

[20] Addressing health inequalities. https://www.manchesterbrc.nihr.ac.uk/about-us/equality-diversity-inclusion/addressing-health-inequalities/ Accessed 15 November 2022.

[21] English indices of deprivation 2019. https://imd-by-postcode.opendatacommunities.org/imd/2019. Accessed 15 November 2022.

[22] What do I need to do? HRA https://www.hra.nhs.uk/planning-and-improving-research/best-practice/public-involvement/what-do-i-need-do/. Accessed 15 November 2022.

[23] Vocal Opportunities. https://wearevocal.org/opportunities/. Accessed 15 November 2022.

[24] Hearing Health Now. https://wearevocal.org/wlrs/listen-up/hearing-health-now/. Accessed 15 November 2022.

[25] Office for National Statistics Census 2011. https://www.ons.gov.uk/census/2011census. Accessed 15 November 2022.

[26] NIHR Manchester BRC and CRF PPIE strategy 2018–2022. https://www.manchesterbrc.nihr.ac.uk/wp-content/uploads/2018/03/BRC-and-CRF-PPIE-strategy-2018-2022-FINAL.pdf. Accessed 15 November 2022.

[27] Manchester City Council Census 2021 Demography https://app.powerbi.com/view?r=eyJrIjoiZjIxNTQ3OWMtNWNmMy00ZWFhLWJhOWQtZTU3Y2IzZGZmZmUzIiwidCI6ImIwY2U3ZDVlLTgxY2QtNDdmYi05NGY3LTI3NmM2MjZiN2IwOSJ9. Accessed 15 November 2022.

[28] Islam S (2020). “We are not Hard to Reach, but we may find it Hard to Trust”Involving and Engaging ‘Seldom Listened to’ Community Voices in Clinical Translational Health Research: A Social Innovation Approach. Res Involv Engagem.

[29] Black Asian and Minority Ethnic Research Advisory Group (BRAG). https://wearevocal.org/opportunities/black-asian-and-minority-ethnic-research-advisory-group-brag/. Accessed 15 November 2022.

[30] Inclusive Research online learning https://courses.manchester.ac.uk/webapps/bbgs-cloud-portal-BB5e13cac4dee6a/app/portal/catalog?locale=en&dir=ltr. Accessed 4 April 2023.

[31] Voice Up. https://wearevocal.org/opportunities/voice-up/. Accessed 15 November 2022.

[32] “We are not hard to reach, we are here, we are visible” https://wearevocal.org/wlrs/listen-up/we-are-not-hard-to-reach-we-are-here-we-are-visible/. Accessed 4 April 2023.

[33] Starling B, Tanswell J (2018). Diversifying audiences and producers of public involvement in scientific research: the AudioLab. Res Involv Engagem.

[34] Planet DIVOC-91 makes a difference. https://wearevocal.org/wlrs/listen-up/visit-planet-divoc-91/. Accessed 23 March 2023.

[35] Radiotherapy &Me. https://wearevocal.org/wlrs/topics/radiotherapy-me/. Accessed 23 March 2023

[36] #MyMSKStory. https://wearevocal.org/wlrs/topics/mymskstory/listen-up/. Accessed 23 March 2023.

[37] The Ideas Fund. https://theideasfund.org/. Accessed 23 March 2023.

[38] The people we work with. https://wearevocal.org/wlrs/listen-up/the-people-we-work-with/. Accessed 4 April 2023.

